# The Internet-Based Intervention Strategies for Empowering Activities in Everyday Life: Qualitative Study of Experiences of Clients With Stroke

**DOI:** 10.2196/56189

**Published:** 2024-08-15

**Authors:** Ida-Maria Barcheus, Maria Ranner, Eva Månsson Lexell, Lars Jacobsson, Maria Larsson-Lund

**Affiliations:** 1 Division of Health, Medicine and Rehabilitation-Occupational Therapy Department of Health, Education and Technology Luleå University of Technology Luleå Sweden; 2 Department of Health Sciences Lund University Lund Sweden; 3 Department of Neurology, Rehabilitation Medicine, Memory Disorders, and Geriatrics Skåne University Hospital Lund-Malmö Sweden; 4 Department of Rehabilitation Medicine Sunderby Hospital Luleå Sweden

**Keywords:** internet-based rehabilitation, occupational therapy intervention, rehabilitation, self-management, stroke, active everyday life, activity-based intervention

## Abstract

**Background:**

There is a need to enhance access to and support for self-management of activities in everyday life after a stroke. Internet-based solutions have the potential to contribute to this development. Consequently, an internet-based intervention called *Strategies for Empowering Activities in Everyday Life* (SEE) was developed. The intervention aims to assist clients in developing management strategies that promote a healthy distribution and balanced engagement in various activities performed in different places and with other people. To further support the development and feasibility of this intervention, more knowledge is needed about clients’ experiences during the intervention process.

**Objective:**

This study aims to explore and describe how clients with stroke experienced the SEE intervention process and whether participation in SEE influenced their experience of everyday life.

**Methods:**

Overall, 9 clients with stroke who received SEE participated in the study—4 (44%) women and 5 (56%) men aged 37 to 73 years. Qualitative interviews about experiences with SEE were conducted twice during the intervention process with each participant. The data were analyzed using the constant comparative method of grounded theory.

**Results:**

The participants’ experiences with the intervention process of SEE formed the core category, conceptualized as *The relevance of and readiness for entering a change process in activities of everyday life differ among clients*, constituting of two main categories: (1) an eye-opener providing agency for a change process and (2) never beginning a change process in activities in everyday life. The results showed that the relevance of and readiness for SEE differed between the participants. The experiences of 78% (7/9) of the participants reflected that the intervention process provided them with an agency to drive their own change process for activities in everyday life to promote health. Overall, 22% (2/9) of the participants refrained from entering a change process during SEE as they did not recognize any need for changes in their activities. When SEE was relevant and adopted as expected, the participants described it as an eye-opener for how they can alter their health based on how they distribute and spend their time on various activities.

**Conclusions:**

SEE has the potential to support clients’ development of self-management and to take an active role in influencing their engagement in activities in everyday life and health. This study identified necessary improvements in the educational program for professionals to enhance delivery and strengthen the therapeutic mechanisms of SEE for future research. To effectively implement internet-based interventions such as SEE, it is crucial to identify clients who express a need for self-management in activities and are ready to invest the effort required to adopt a change process. Furthermore, it is indicated that participants’ self-analysis of their everyday activities empowers them to adopt new self-management strategies, which can also benefit other interventions.

## Introduction

### Background

People with stroke can experience unmet rehabilitation needs when they try to return to active everyday life after a stroke [1], which can lead to further deterioration of health [1,2]. Therefore, rehabilitation interventions focusing on life after stroke need to be developed [3]. Interventions supporting self-management that empower clients to use their own skills and internal and external resources are important for reducing the impact of strokes on everyday life [4,5]. In addition, being able to manage and adapt to a life on new conditions after stroke can be facilitated when aspects related to engagement in activities in everyday life are promoted rather than remediation of impairments [6,7]. However, access to rehabilitation after stroke is insufficient and geographically unequally distributed [8,9]. The design and provision of new internet-based interventions can fill this gap and act as catalysts for the needed development [10,11].

Several eHealth solutions exist that are applied for clients with stroke [12-14], often with digital components embedded within a team-based rehabilitation intervention. Reviews [12,13] show that digital solutions often focus on assessments and restorative training. However, to our knowledge, internet-based rehabilitation interventions that cover all steps in the rehabilitation process and at the same time support clients’ self-management of activities in everyday life are scarce. Therefore, a new internet-based and person-centered intervention focusing on facilitating self-management, “*Strategies for Empowering Activities in Everyday Life*” (SEE; version 1.0), was developed [15,16]. SEE aims to support an active everyday life, enabling a client to engage in a range of activities performed in different places and with other people [16]. Furthermore, SEE supports the client in developing management strategies that enable a healthy distribution of daily activities. The program theory of SEE [16] is based on knowledge of how reflections of and doing activities in everyday life can be both a therapeutic means and an end in a change process [17,18]. The internet-based design of SEE facilitates clients’ active distance learning [19,20] using web modules with short video clips with subsequent assignments and reflections. The clients go through the web modules before being guided during dialogues with occupational therapy professionals who aim to further support clients’ analysis, reflection, and development of self-management skills. Thereby, clients can take on an active role, and professionals’ resources can be focused on supporting the clients’ change process rather than informing and giving advice.

### Objectives

Due to the novelty of SEE, there is a lack of knowledge about how clients experience the intervention process. Thus, additional knowledge about the facilitators, hindrances, and therapeutic mechanisms experienced during this process is warranted to further support the development and feasibility of SEE. Such experiences can provide new insights that are important for optimizing the design, content, and delivery of internet-based interventions and remove barriers to participation for clients with disabilities. The aim of this study was to explore and describe how clients with stroke experienced the SEE intervention process and whether participation in SEE influenced their experience of everyday life.

## Methods

### Design

This study used a qualitative design with a grounded theory approach in which constant comparative analysis of interview data and memo writing were conducted [21]. This method was chosen because it is suitable for inductively discovering patterns in interactions and processes, such as when people enter a process of change in their activities in everyday life. To follow experiences of the intervention process, each participant was interviewed at 2 different occasions over time. This study is part of a larger project evaluating the feasibility of SEE [15], which follows the Medical Research Council guidance for development and evaluation of complex interventions [22]. To ensure transparency and quality in reporting the study, we adhered to the Standards for Reporting Qualitative Research [23].

### Researchers’ Reflexivity

To enhance and maintain the authors’ reflexivity [24,25] during the research process, regular discussions concerning data collection, data interpretations, and writing occurred within diverse author constellations. While only 1 author met the participants (during data collection), the remaining authors had no relation to them. The authors, each possessing extensive clinical and research backgrounds in rehabilitation, had varying experiences with interventions in occupational therapy and neuropsychology across different settings for clients with stroke. In addition, their diverse roles in developing SEE—some were not engaged or remained on the periphery—prompted critical self-assessment of their perspectives, assumptions, and biases. This deliberate reflexivity aimed to improve the overall quality of the study results.

### Participants and Study Context

The potential participants were recruited for a feasibility evaluation research project of SEE [15] from databases including people admitted to hospitals for stroke in the uptake area of 4 hospitals in northern Sweden. The inclusion criteria for participation in the feasibility project were (1) stroke onset between 3 and 24 months, (2) age of 18 to 75 years, (3) moderate disability or good recovery based on the Glasgow Outcome Scale–Extended [26], (4) ability to express themselves verbally and in writing, (5) access to the internet (computer, tablet, or phone that they were able to use), and (6) motivation to change activities in everyday life. Their motivation [27] to change was explored through a set of questions investigating whether daily activities were a problem and whether they were currently ready to implement a change. To be included, the potential participants had to respond positively to all the questions regarding their motivation. The exclusion criteria were (1) other illnesses or injuries affecting daily activities and (2) depression based on the Hospital Anxiety and Depression Scale [28]. The choice of selecting those with moderate disability or good recovery according to the Glasgow Outcome Scale–Extended (inclusion criterion 3) was based on knowledge [29] that those with more severe disability often have more difficulties with managing technology independently.

In total, 15 clients agreed to participate in the ongoing feasibility evaluation [15]. In this qualitative study, the following additional inclusion criteria were applied: having completed the first 3 phases of the intervention process for SEE in accordance with the established guidelines and not receiving a similar intervention in parallel to SEE. Of the original 15 participants in the feasibility study, 3 (20%) dropped out (1/3, 33% entered inpatient rehabilitation and 2/3, 67% declined without giving reasons) and another 3 (20%) participants’ intervention process did not adhere to the SEE guidelines, leaving 9 (60%) participants who received written and oral information about the qualitative study. Before the qualitative interviews, all participants met the researchers for evaluation of activity and health variables at baseline and after 4 months as part of the feasibility project [15]. At the time of the second interview, 1 participant had received inpatient rehabilitation and, therefore, did not meet the second additional inclusion criterion. Consequently, only the first interview with this participant was included in the study. The sociodemographic data (Table 1) showed that the participants were mostly employed and all had a good recovery after their stroke [26] at the time of the intervention.

**Table 1:** Characteristics of the participants with stroke taking part in the internet-based intervention Strategies for Empowering Activities in Everyday Life (n=9).

Characteristic		Values
**Sex, n (%)**		
	Female	4 (44)
	Male	5 (56)
Age (y), mean (SD; range)		59 (12.7; 37-73)
Time since injury (mo), median (range)		6 (7.57; 3-25)
**Severity of disability, n (%)**		
	Good recovery	9 (100)
**Educational level, n (%)**		
	Primary school	1 (11)
	Upper secondary school	4 (44)
	University	4 (44)
**Employment status, n (%)**		
	Working full time	1 (11)
	Working part time	4 (44)
	Retired	4 (44)
**Living condition, n (%)**		
	Married or cohabiting with children not living at home	6 (67)
	Married or cohabiting with children living at home	2 (22)
	Single	1 (11)

### The Intervention

SEE [15,16] is available through the national health care platform in Sweden, called 1177, and is delivered in 4 phases [16]. The first 3 phases take approximately 1 month to complete and end with the establishment of an activity plan. In the fourth phase, the activity plan is implemented for an additional 2 to 4 months depending on individual needs. The first phase of SEE supports clients in reflecting and evaluating their current engagement in activities in everyday life and their use of strategies to manage those activities. The second phase includes a web program with modules promoting the clients’ development of knowledge about activities, self-analysis, and sustainable strategies in everyday life and for health. After completing the web program, in the third phase, the client establishes an activity plan with goals and associated management strategies based on the knowledge and experience developed during the earlier phases. The activity plan aims to guide the forthcoming change process. Finally, in the fourth phase, the client’s implementation of management strategies to achieve goals in activities in everyday life based on the activity plan is supported. During all phases of the intervention process, the client works in close collaboration with an occupational therapist (OT), who guides and supports the client’s self-reflection and changes in activities. The guidance occurs through video meetings or as written feedback after each module in accordance with the intervention guide of SEE. A total of 4 OTs delivered SEE to participants in this study. Because the intervention is new and is included in a feasibility project, the OTs were also new users of SEE. A more detailed description of the 4 phases and the program theory underlying the content and delivery, as well as of SEE’s educational program for the deliverers, can be found in previous studies [16].

### Data Collection

The data were collected through qualitative interviews [30] at 2 time points for each participant with the aim of providing an understanding of their experiences of the intervention process over time. The first interview was conducted 2 weeks after the activity plan in SEE was established (approximately 6 weeks after the start of SEE), and the second interview was conducted 4 to 5 months after the participant had entered SEE. Of the 9 participants, 7 (78%) had not completed SEE at the time of the second interview (the intervention process was ongoing), and the other 2 (22%) participants had reached the goals in the activity plan and completed SEE before the second interview. The interview guide during the first interview focused on participants’ experiences of everyday life after the stroke, before starting SEE, and experiences of the intervention process so far. The second interview focused on whether participants tried to implement changes in their life and whether they had developed new strategies to manage activities in everyday life based on what they had learned during the program. Follow-up questions such as “tell me more...” and “can you describe...” were used in both interviews to obtain more descriptions and in-depth clarifications. The interview guide was refined during the data collection and analysis process, which meant that the second interview with each participant picked up where the first one left off. The interviews were conducted between December 2020 and February 2023 via videoconferencing (by IMB), recorded as audio files, and transcribed verbatim.

### Data Analysis

Data analysis started immediately after the first interview was conducted, and the guidelines of the constant comparative method with continuous memo writing were used throughout the analysis process [21]. In the initial coding, the transcribed interviews were read to gain an overall understanding of the participants’ experiences of the intervention process. The content of each interview provided insights and enriched future interviews, especially the next interview with the same participant. The transcripts were then analyzed through open coding, where the author (IMB) identified units of meaning that corresponded to the aim of the study. Questions focused on discovering events, interactions, processes, and their meanings based on the aim helped the author form codes. Codes that pertained to similar meanings and experiences were sorted into preliminary categories. An example from this step in the analysis process shows how a unit of meaning is sorted in a preliminary category. “When you get questions and when you talk and reflect on different things [activities in everyday life] you always get new things to think about” was given the code “new insights,” which was further sorted into the preliminary category “Seeing everyday activities in a new light.”

These preliminary categories were then scrutinized and discussed based on the transcripts with the coauthors (MR and MLL). An understanding of how the participants experienced the SEE intervention process and how they managed everyday life formed working metaphors of *seeing* and *doing changes*. The metaphors were further used in axial coding (IBM, MR, and MLL), where the preliminary categories reflected experiences from different points in time that could be linked to form subcategories. In this analysis step, 2 main categories with 1 to 3 categories and associated subcategories were identified. Finally, the core category *The relevance of and readiness for entering a change process in activities of everyday life differ among clients* emerged to conceptualize the essence of the participants’ experiences of SEE. In identifying the core and main categories, the authors moved to a conceptualization of data at a higher analytical level in accordance with the constant comparative method.

Throughout the analysis process, constant comparisons were conducted among preliminary categories, codes, transcribed text, and memos. This iterative approach aimed to enhance reflexivity [24] and ensure the quality of the analysis. The authors (IBM, MR, and MLL) played different roles throughout the analysis process, so one step made by one researcher was discussed with the others to ensure that the analysis was based on the data. The other authors (EML and LJ) asked critical questions about the interpretations based on the transcripts during the last steps of the analysis to increase the credibility [31] of the results. In presenting the results, quotes were carefully chosen to validate the analysis.

### Ethical Considerations

This study was approved by the Swedish Ethical Review Authority (2019-04993). All participants provided written and verbal informed consent to take part in the study. The interview transcripts were deidentified, and each participant was assigned a unique ID number. The participants took part in the intervention and research without any cost or compensation.

## Results

### Overview

The analysis of the participants’ experiences with the SEE intervention process formed the core category *The relevance of and readiness for entering a change process in activities of everyday life differ among clients*. The core category included 2 main categories with categories and subcategories (Textbox 1) that reflected the participants’ divergent experiences of SEE. For participants in one main category, over time, SEE evoked a change process in activities in everyday life (7/9, 78% of the participants), whereas for participants in the other main category, no change process occurred (2/9, 22% of the participants).

**Overview of the results of the experiences of the intervention process in Strategies for Empowering Activities in Everyday Life in the participants with stroke.**

**Core category: the relevance of and readiness for to enter a change process in activities of everyday life differ among clients**

- Main category: an eye-opener providing agency for a change process
  Category: dealing with a less active everyday life
  Category: reaching new insights leads to a new direction for change
    - Subcategory: discovering the potential of changes in everyday life through learning and self-analysis
    - Subcategory: exploring possibilities for managing activities by going from thought to action
  Category: establishing a new mindset and attitudes to self-manage everyday life for health
    - Subcategory: striving to reach goals
    - Subcategory: achieving control and having established a new way of managing activities in everyday life
- Main category: never beginning a change process
  Category: nothing has changed in everyday life

### Main Category: An Eye-Opener Providing Agency for a Change Process

#### Overview

In total, 78% (7/9) of the participants experienced the intervention process of SEE as an eye-opener for how they could alter their health based on how they distributed and spent their time in various activities and through the management strategies they adopted. This meant that, by participating in SEE, they developed their competencies as they gained new knowledge and experiences in monitoring and managing activities. This enabled them to assume an active role in which they pushed their own change process forward toward an active everyday life. The main category included 3 categories that reflected how the participants’ experiences evolved in a chronological order from the onset of stroke until the last interview 4 to 5 months after entering SEE.

#### Category: Dealing With a Less Active Everyday Life

This category describes the participants’ experiences of discovering restricted possibilities to engage in everyday life after their stroke and how they struggled with managing these before entering SEE.

Participants described how, after the stroke, they gradually discovered difficulties when trying to return to old habits and routines for their engagement in activities in everyday life. Activities that previously had been performed without much effort or reflection were now, after the stroke, described as physically and cognitively demanding and taking more time to perform. They had to deal with loss of body functions, problems with memory, fatigue, and communication difficulties that often involved having to also handle emotional reactions. These challenges were described as most difficult to manage during activities in society and when interacting with others, such as shopping, socializing, or working. They were engaged in fewer activities than before the stroke, and much of the time was spent in sedentary activities. They said that they were less active than they were before the stroke, and they were dissatisfied with how time was spent, as described by one participant:

I felt I had been stuck on the TV sofa a bit too much. If I take a break on the sofa in the afternoon, I’m still there for several hours...I wanted to get away from that...

Participant 7; first interview

The participants described how they struggled to overcome their impairments and, thereby, their engagement in activities in everyday life, that is, how they trained various body functions through physical exercises or how they trained while doing activities (eg, using both hands when eating or training memory by doing crosswords). They described how they tried to manage everyday life by avoiding or omitting activities and by allocating time for rest to recover from the stroke. One participant said that “I have not been able to do much or have not dared to do much*”* (participant 1; first interview). During this phase, the participants perceived a lack of professional support, and they felt, to various extents, abandoned and uncertain when dealing with their new life situation. Fear of causing a new stroke or deterioration of body functions was expressed. They said that they acted to the best of their knowledge but it was not enough to reach an optimal and satisfying everyday life and they were interested in alternative solutions.

#### Category: Reaching New Insights Leads to a New Direction for Change

##### Overview

This category consists of 2 subcategories that describe the participants’ experiences of how the modules in SEE, including both the web program and the guiding dialogues with the OT, developed their knowledge, self-analysis, and management strategies in everyday life. During the first 6 weeks of SEE, they discovered new needs and possibilities in their activities of everyday life and started to plan and act for a change in a new direction.

##### Discovering the Potential of Changes in Everyday Life Through Learning and Self-Analysis

The participants’ experiences showed how the knowledge gained from the video modules, including the related self-analysis assignments and reflections, improved their ability to understand their current situation and their potential to change everyday life. Several new perspectives and insights into activities in everyday life that they had not thought about before became evident in the modules, as expressed by one participant*:*

I think I have reflected a lot on things and it has sometimes been difficult to answer the questions [the assignments in the SEE modules]. Sometimes I thought...the same question as last week...you see it from different angles, and then you become reflective and from that, new ideas are born.

Participant 1; first interview

Participants’ broader reflection on everyday life meant that they also began to realize that what they do (ie, which activities they engage in) and not only their underlying capacity influences their health. They had come to realize that the way in which they engaged in some activities limited their health and that they lacked activities in life that could contribute to recovery. The participants also described how the modules that presented alternative ways of doing different activities had made them reflect on how they themselves could manage their activity problems. This experience was expressed as an eye-opener, as illustrated in the following quote:

You think about what you are doing, when you go through the modules, it is thought-provoking and then you can understand things better.

Participant 9; first interview

The participants’ experiences showed how they started to develop their ability to reflect and analyze their activities. Self-analysis of the time they spent on various types of activities each day or week increased their awareness of situations in everyday life that could become challenging or restricting. The analysis led them to talk about the importance of doing more activities with others and doing more activities outside the home. Starting to think more about societal and social aspects of activities together with physical aspects was common among the participants, as illustrated in the following quote:

When you assess [in the assignments in SEE’s modules] how you feel about doing different things, whether you are satisfied or not satisfied, I am not satisfied with what I do physically, I am not truly satisfied with how my social life looks like now...It has made it clear to me, that this is how it is right now and what I can do to make it better.

Participant 5; first interview

Similarly, self-analyses about the different efforts (physical and mental) of engaging in various activities and the distribution of these activities were important. These experiences contributed to new insights that facilitated their acceptance of their changed capacity. This meant that their former self-evident everyday life needed to be renegotiated. In this regard, guiding dialogues with OTs were described as important resources. One participant shared his dialogues with the OT:

When you get questions and you talk and reflect on different things [with the OT]...it is always good...that you get new things to reflect on and think about, what do I want to do and how should I do it.

Participant 9; first interview

Taken together, the self-analysis and reflections supported the participants in thinking about what they could change or not, which made them realize the need to prioritize and plan activities more consciously to improve their situation and health.

##### Exploring Possibilities for Managing Activities by Going From Thought to Action

The participants’ descriptions reflected that early in the intervention, they already had started to explore their potential and make changes in their activities between the different modules. On the basis of their new thoughts (from the analysis), they quickly realized a need to manage everyday life differently to not compromise their health. One participant said the following:

[I should] not take on a lot of stuff, even though it is fun, it gets troublesome...now that I do not have the same capacity...now I have begun to accept that I can rest; I think this calendar will be good, where I can see that I have actually done a lot so now I do not need to do anything more today.

Participant 1; first interview

Their experiences showed that they were observant about what happened when they carried out changes in their engagement in activities. When they met the OT, these experiences were discussed and evaluated to further support modifications or additional changes in their everyday activities. Through this approach, they learned to better understand their capacity and possibilities. During this process, the participants started to prioritize and act more accordingly with their own needs and desires, adding a greater variety of activities and actively seeking activities outside the home to do together with others. This also meant that the distribution of various activities was reconsidered. One participant who started to reduce and redistribute social activities said the following:

And make sure that they [social activities] are a maximum of two times per week...then I have to see what I can do, to manage, and to be able to fully enjoy the opportunity and to recover.

Participant 5; first interview

The participants described that establishing an activity plan with goals and strategies for change in dialogue with the OT was a way to summarize and clarify for themselves what is important in everyday life for their well-being and how to achieve it. They described the activity plan as a guiding tool or a road map that was reasonable and would support them moving forward. One participant described the plan during the first interview as follows:

The plan was to avoid scheduling too many [activities] in the same week.

This participant elaborated a bit later in the interview:

Yes, but it is very positive because by putting your situation into words, how you feel and how you want it to be, you can control...yes, this is how I want it [with my activities in everyday life] instead of wanting to feel better, yes, what is it to feel better, what steps should you take to get there and what is reasonable.

Participant 5; second interview

#### Category: Establishing a New Mindset and Attitudes to Self-Manage Everyday Life for Health

##### Overview

This category consists of 2 subcategories that reflect the participants’ experiences of their change process that continued to evolve from 2 weeks after the establishment of the activity plan until the second interview. Participants’ experiences showed how they continued to learn during their implementation of changes, leading to a new mindset and attitude toward activities in everyday life. At the second interview 4 to 5 months after the start of SEE, most participants had implemented the plan and achieved their goals.

##### Striving to Reach Goals

The participants’ experienced that the work of implementing the activity plan meant that they continued their explorative learning process while changing their everyday activities in accordance with the plan. They were observant of when they deviated from the planned changes in activities and noted the negative consequences. The process was iterative as they sometimes fell back into old habits and had to struggle to stay on track and continue with their changes in accordance with the plan. One participant said the following:

I still fall back into it [doing a lot at the same time], and I made this plan that when I’m at work, [I will do] one thing at a time, close down the email after I have checked it in the morning and it is hard as hell!

Participant 5; second interview

This learning process built new insights into what was working or not and how their choice of acting either promoted or compromised their health. The participants described the activity plan as a guide forward throughout the process that, together with the follow-up meetings with the OT, facilitated the participants’ progress. They had received confirmation from the OT and felt safe continuing the change process. Most participants kept the plan throughout the process without any changes, but some said that fulfilling the plan meant that they came to new insights about their capacity and possibilities. These insights included becoming aware that their goals were unrealistic and contributed to them striving for something that had a negative impact on their health.

##### Achieving Control and Having Established a New Way of Managing Activities in Everyday Life

The participants’ experiences showed that, at the time of the second interview, they had learned how to manage activities to stay active and healthy. Their experiences reflected how they continuously monitored and analyzed their activities to discover any need for adaptations in their way of managing everyday life during a day or week or from a more long-term perspective. This empowered them to achieve control and make decisions to steer everyday life toward a healthy distribution of activities based on what was best for them. Previous fears of engaging in different activities and risk of deterioration had disappeared as they had developed confidence in managing forthcoming changes. Being able to actively verbalize and act upon their needs gave them a sense of having control in everyday life, as illustrated in the following quote:

I met with the occupational health service, and they said there was a difference...because then [before SEE] I just sat and hung with my head; I was not involved in discussions, unlike now when I met them just a few weeks ago, and I talked a lot more than I did before.

Participant 2; first interview

The participants said that they had established new habits in everyday life that facilitated their possibility of being active in a way different from before SEE. They had achieved a more balanced distribution of various activities for what they wanted and needed to do in everyday life. Some that previously engaged in only a few activities, mainly in their home, had established habits for doing several new activities in various places together with others in society. Others had reduced their engagement in different social contexts to make more time for new activities that contributed to relaxation and recovery. Furthermore, activities associated with high physical and mental demands were redistributed over the course of the week to achieve a better balance. One participant said the following:

Before I worked with the modules [in SEE], I had a lot of stress; I wanted to finish everything immediately. Now I know I must take my time and do little a time [activities]. The modules have helped me to find strategies to be able to do everything I want without stress, which is good for my health.

Participant 6; second interview

The participants’ experiences showed that, in addition to changes in activities, it was common to adopt a healthy lifestyle in general related to physical activity and meals. They talked about engaging in new activities that were simultaneously socially stimulating and physically demanding as natural parts of everyday life, as illustrated in the following quote:

Going to dance is a goal for the future. There you can meet a lot of people. You always have small goals, but I have to exercise so I get better balance...but of course, if you do it [go to dance] you get a better and better balance.

Participant 9; second interview

They established a mindset focused on everyday activities that promoted their well-being and developed a new attitude toward making changes in activities. One participant said the following:

I will continue using all the tips I got from the program. I think it [the SEE] is great for moving forward in life.

Participant 6; second interview

### Main Category: Never Beginning a Change Process

#### Overview

The experiences of the 2 participants who completed SEE without beginning a change process reflected that nothing had truly changed in everyday life before or after the intervention process.

#### Category: Nothing Has Changed in Everyday Life

The participants’ experiences showed that their participation in SEE was driven by curiosity and whether the new intervention could add something to their lives. Being a part of the research project was also described as a way to pass time and as a way to contribute to the development of health care. They did not identify any loss of or unsatisfactory engagement in activities of everyday life before, during, or after SEE and did not identify any changes they wanted to achieve. Their experiences reflected that they had not increased their knowledge or made practical use of the content of the modules and they were not able to recall their activity plan. One participant emphasized the importance of support with physical training in activities after a stroke and the lack of it in SEE. In the second interview, both participants said that nothing had truly changed in everyday life but they felt safer and confirmed their situation, as illustrated in the following quote:

You get a different perspective on the whole thing, and you are confirmed that you may be doing things well, and that also gives you a sense of security in your rehabilitation. Because if you must do everything on your own, you might get lost.

Participant 4; second interview

## Discussion

### Principal Findings

This study focused on how people with stroke experienced the intervention process of SEE, a new internet-based intervention that promotes an active and healthy everyday life. The results showed that the relevance of and readiness to go into the intervention process differed between the participants. This was evident as the 2 main categories showed that some participants (7/9, 78%) experienced that they entered a process of change in activities of everyday life, whereas the others (2/9, 22%) experienced no need to change their activities and no process of change. When SEE was adopted as expected, the participants experienced that it provided them with an agency to drive their change process toward improved health. During this process, they established new habits and more efficient ways of managing their activities in everyday life that also contributed to a sense of control and confidence that they could master their situation.

### Comparison With Prior Work

This study highlights the importance of identifying people who express a need to make changes in activities of everyday life and who are ready to invest the effort needed to adopt a process of change if SEE is to be successfully implemented. Both relevance and readiness are fundamentally important for the motivation to initiate and manage a change process [27,32]. In addition, social and environmental aspects are important to consider to fully understand the conditions influencing behavior change [33]. The importance of detecting the participants’ motivation and conditions for change was part of the inclusion criteria and was also addressed in the first phase of SEE [16]. Nevertheless, 22% (2/9) of the participants went through SEE without truly identifying any need for change. The results showed, similarly to other research [34,35], the difficulty of identifying and facilitating different conditions and levels of readiness for change. The results of this study indicate the need to scrutinize the inclusion process and develop the educational program for professionals in future research to identify clients who will respond to SEE. On the basis of the transtheoretical model of behavior change [27] in the program theory of SEE [16], the professional’s knowledge of how to conduct dialogues using motivational interviewing [32] to explore readiness for change needs to be improved. Through the development of the educational program, professionals can become better prepared to guide clients to other measures or interventions instead of or before SEE when needed. All interventions can be expected to have both responders and nonresponders. Therefore, interventions should include tools to identify those who are responders or nonresponders who need another more suitable alternative intervention in a timely manner. This can enhance person-centeredness when the right interventions can be offered at the right time for each person.

For persons who experience readiness to learn how to self-manage their everyday life and health in new ways, it is indicated that SEE can contribute. In line with this, the Stroke Action Plan for Europe [3] highlights the need for developing self-management support for life after stroke. The results showed that 78% (7/9) of the participants in the internet-based SEE took on an active role and drove their change process forward. Previously, digital solutions have often been used to augment stroke rehabilitation, but knowledge about clients’ ability to invest in self-management programs through the internet has been unclear [36]. To our knowledge, this is the first completely internet-based intervention that combines clients’ own work in web modules with web-based guiding sessions with an OT. The results, which reflect the qualitative aspect of testing SEE in the feasibility phase, are promising for continuing to improve access to rehabilitation focusing on health rather than disability after the subacute phase of stroke. The participants’ experiences show how, initially, before SEE, they tried to deal with a less active life by focusing on restoring underlying functions by engaging in physical exercise. These results indicate that participants acting on their own were influenced by the typical rehabilitation people receive after stroke, rehabilitation that focuses on improving functions [37,38]. Gradually, through SEE, the participants established a new mindset and learned a way of self-managing their everyday life. This highlights the need to introduce self-management earlier in the recovery process after stroke [36], also demonstrated by the participants in this study, where SEE helped them avoid a downward spiral of deterioration of their health. The results show how the participants developed skills to self-manage, which empowered them to take control of their new life situation, something that has also been similarly described in other studies of non–internet-based self-management programs for people with stroke [36,39].

At the end of the change process, 78% (7/9) of the participants experienced SEE as an eye-opener and had established new sustainable habits, including regular monitoring and managing activities in everyday life, to promote health. This influenced them to reach a balanced engagement and a healthy distribution of a variety of activities in everyday life, enabling a more active life. This reflects the strength of the activity-based approach of SEE, in line with the findings of other occupational therapy interventions [40-42] showing the value of using activity as a therapeutic agent in a change process. A common component of these interventions, including SEE, is the use of self-analysis of activities in everyday life in which the client identifies the need for changes, which in turn facilitates the goal-setting procedure. However, with the use of web modules and assignments as in SEE, the participants work on their own, analyzing their activities from different perspectives, which seems to make them better prepared for the dialogues with the OT. This approach has the potential to lead to more targeted and efficient dialogues supporting clients in their own change processes.

Several important therapeutic mechanisms seem to be involved in the explorative learning process in activities of everyday life that influence a successful intervention. The results show how an iterative ongoing process developed a therapeutic mechanism in terms of self-reflection and self-monitoring of everyday activities. This process formed a motivating and empowering element and a base for testing and adopting new self-initiated strategies during the participants’ engagement. Together, these therapeutic mechanisms seem to have contributed to an eye-opener and literacy regarding the role of activities in everyday life concerning health, a relationship that otherwise can remain unknown or taken for granted and, therefore, not consciously addressed as a resource in self-management. The increased literacy related to health and increased empowerment are 2 closely linked components needed to support people in becoming effective self-managers [43]. These components reflected the experiences of the 78% (7/9) of the participants who entered a change process through SEE. Importantly, the participants learned to know their capacity and possibilities better concerning how their engagement in and distribution of activities could both promote and compromise their health. Improved literacy about how activities can affect health in both directions, either promoting or compromising health, has also been found to be important in lifestyle programs [44,45]. This result indicates the importance of delivering SEE with the competence to apply the models of human activities [16] that are included in the program theory of SEE as a therapeutic means in coaching dialogues with clients. Thus, knowledge about the therapeutic mechanisms of SEE and how activities as a means and end for change are reflected in the experiences of the 78% (7/9) of the participants can further contribute to developing SEE’s educational program for professionals.

The results of this study also show how the distance pedagogic format of SEE [16] can provide new advantages and new therapeutic mechanisms that are not available in the traditional format of rehabilitation. This indicates the importance of making interventions related to the expertise of different professions available on national health care platforms such as 1177 in Sweden. As SEE is the first internet-based intervention in occupational therapy, it can provide OTs with a digital solution that can either be an “add on” and complement existing interventions or work as a single intervention. New solutions provide a greater variety of alternatives, and as reflected in the results, it is important to choose a relevant intervention and meet the needs of each client.

The participants’ experiences reflected how the flipped-classroom methodology [19,20] supported their active learning. The importance of allowing people to work independently to facilitate their own drive to develop new strategies has been emphasized [46]. Such a design can further increase the person-centeredness of the delivery of interventions and improve the motivation for change [32,46]. The results also showed how the establishment of an activity plan, in line with the findings of other studies [34], was important for the participants. The content of the activity plan (including a clear link among needs, goals, and strategies) clarified what was important in the participants’ everyday lives for their well-being and guided them forward to achieve it. Thus, the flipped-classroom design in combination with the establishment of the activity plan can be considered important ingredients to support the client in taking responsibility in their own change process while at the same time helping the professional to work in a person-centered way. This knowledge about how we can take advantage of a distance-based format can be valuable when developing other interventions and rehabilitation practices.

### Limitations

Qualitative interview studies can contribute to an understanding of the experiences and therapeutic mechanisms of complex interventions that are important for identifying the need for improvements before conducting efficacy studies [22]. The numbers presented in the findings should not be interpreted statistically due to the nonrandomized selection of participants and the openness of the data collection method. All the participants had a good recovery after their stroke, which limits the transferability of the results to the general population of people with stroke. A more diverse level of severity of disability among the participants would have been preferable, but the number of people who tried and completed SEE was limited. The other sociodemographic variables (such as sex, educational level, and employment status) were well distributed among the participants. It is also important to note that the data were based on participants who completed or were about to complete SEE. Thus, those who chose to drop out were not asked to participate for ethical reasons. It is also important to consider that the participants’ experiences were influenced by the fact that their OT was not previously experienced in applying SEE. As the participants were among the first clients with whom OTs implemented SEE, this might have impacted the quality of delivery. A focus group study of OTs’ experiences [47] has also confirmed that they had to go through a transition from ordinary practice to learn how to deliver the internet-based SEE. The authors’ reflexivity [24,25] and the trustworthiness [31] of the study were enhanced through continuous discussions among the authors in various constellations throughout the research process.

### Conclusions

The participants’ experiences reflected that the relevance and readiness of the intervention process in SEE differ among participants. When SEE is relevant and adopted as expected, it can support clients’ development of self-management and take an active role in influencing their engagement in activities in everyday life and health. SEE can provide clients with an agency to drive their change process forward and establish new habits with more efficient ways of managing and mastering their situation. The results indicate the importance of identifying people who express a need to develop self-management strategies for activities of everyday life and who are ready to invest the effort required to adopt a change process. This readiness seems to be crucial for the successful implementation of internet-based interventions such as SEE. The participants’ own analysis and reflection of their everyday activities served as a motivating and empowering element, providing a foundation for adopting new self-management strategies. This element, together with the distance-based design of the intervention, may be a valuable therapeutic mechanism that can be adopted in other interventions striving to encourage participants to drive their own change process. Furthermore, this study identified necessary improvements in the educational program for professionals to enhance delivery and strengthen the therapeutic mechanisms of SEE for future research.

## Abbreviations

OT: occupational therapist
SEE: Strategies for Empowering Activities in Everyday Life

## Data Availability

The data sets generated during and analyzed during this study are not publicly available due to Swedish ethical and legal restrictions under the Swedish Act for Ethical Review of Research on Humans and the Swedish Secrecy Act but are available from the Luleå University of Technology on reasonable request. The data are archived at Luleå University of Technology in accordance with Swedish law and regulations, and requests to access data can be sent to the email id mentioned on the Luleå University of Technology web page [48].
